# Duality of the Brain—Mind Reading

**Published:** 1887-06

**Authors:** 


### DUALITY OF THE BRAIN—MIND READING EXPLAINED.

The paper read by our Managing Editor at the Georgia Medical Association, entitled "Duality of the Brain ; a Theory of Mind-reading, Slate-writing, etc." has led to considerable inquiry, with requests for its publication by two or three parties—one being an editor of a scientific journal. The paper is held for the Transactions of the Medical Association.

The following abstract of the paper, published in the *Atlanta Constitution*, gives a brief outline of its leading points :

Dr. R. C. Word, editor of the SOUTHERN MEDICAL RECORD, read a paper entitled The Duality of the Brain, with a Theory for Mind-reading and Slate-writing.

"It appeared that the Doctor's idea was that a man has two brains, which, under certain peculiar circumstances, may and do act separately and, sometimes, without a consciousness of the fact as between the two brains. Ordinarily the two brains, in their normal mental operation, act as one, but may act separately as in cases, not uncommon, wherein the crazy man knows that he is insane. In this case the one side of the brain is sound and the other side is unsound. Cases were mentioned wherein a person was subject to peculiar fits or mental states, and when in this peculiar condition the man seemed to be a different individual from his ordinary self, having no recollection of things which occurred in his normal or ordinary state, and remembering the incidents that occurred during his abnormal state only when the fit or spell was on him.

"Mind-reading, according to Dr. Word's theory, is a phase of mesmerism. The mesmerized man is in what he styles an electro-negative or passive condition. There are, probably, 20 per cent. of mankind who are constitutionally electro-negative. Such are very easily mesmerized, and perhaps there are 20 per cent. of people who are partially electro-negative, and who, by practice and perseverance, may be brought under the mesmeric influence. Females are more susceptible than males.

"The more frequently a person is mesmerized the more sensitive and susceptible he becomes, so that he may even throw himself into that state, and voluntarily become electro negative to any one with whom he is *en rapport ;* the word 'rapport' meaning contact or in position where a nerve or magnetic current may flow between the parties. It is possible for a number of persons to be *en rapport* at the same time.

"The mind-reader is an electro-negative subject, who, by practice, has become exceedingly sensitive to impressions so that he is liable to be influenced by any one with whom he comes in contact, especially if the person whose mind he seeks to read will concentrate his thoughts upon any particular object by which he becomes more positive, and the more easily influences or dominates the other who is mesmerized, or at least passive or recipient in his nervous relation to the person who is positive.

4

"Dr. Word said there were many grades or classes of the mesmeric state. Some were in a hypnotic or unconscious condition; some were partially so, and others were fully awake and yet impressible and subject to the mesmeric influence. He expressed the strange and novel position that one side of the brain might be electro-negative to the other side. so that a kind of intercourse might exist between them; the one side conversing with the other side, so to speak This, he said, really occurred with the slate-writer. Thoughts, incidents and latent memories from one side of the brain are written automatically and seem to the other, or conscious side, to come from an outside or third party.

"Where a circle, consisting of a number of persons, is formed the electro-negative subject, called by the Spiritualists a 'Medium,' may get thoughts or impressions from anyone in the circle, and if only one side of the brain is electro-negative his own thoughts are liable to mingle with the thoughts of the others, and be automatically written. If both sides of his brain are electro-negative he falls into a hypnotic or trance condition, which is the same as the deeper phases of the mesmeric condition.

"Upon the subject of whether the spirits can or do communicate through an electro-negative party, the Doctor was rather non-committal. He said that he would not deny its possibility; he thought the disembodied spirit could not communicate with the material world unless by entering and using the brain of a living organism. As the organist manipulates the keys of his instrument to dispense melodious sounds, so the soul of man uses the brain and nervous system to communicate with his fellows; and as the soul is immaterial and may pass into and through solids, so the disembodied spirit may enter and enthrone itself in the brain of a living subject, and thus be able to see and communicate with the physical world. Having thus entered and gotten control of the brain, it could, perhaps, impress the party in the same way that a mesmerizer impresses the subject mesmerized, causing him to think, feel, see, and believe anything he desired—even, in some cases, to hear clairaudiantly, to see clairvoyantly, and to see or imagine the presence of materialized forms.

"The paper was listened to with great interest."

